# The Use of Visual Analysis for Gait and Foot Posture in Children with Developmental Dysplasia of the Hip

**DOI:** 10.3390/diagnostics13050973

**Published:** 2023-03-03

**Authors:** Veronika Vasilcova, Moqfa AlHarthi, Ayman H. Jawadi, Martin Zvonař

**Affiliations:** 1 Department of Kinanthropology, Faculty of Sport Science, Masaryk University in Brno, Kamenice 753/5, 62500 Brno, Czech Republic; 2 Pediatric Rehabilitation Department, King Abdullah Specialized Children’s Hospital, P.O. Box 22490, Riyadh 11426, Saudi Arabia; 3 College of Medicine, King Saud bin Abdul-Aziz University for Health Sciences, P.O. Box 22490, Riyadh 11426, Saudi Arabia; 4 Division of Sport Motorics and Methodology in Kinanthropology, Department of Kinesiology, Faculty of Sport Science, Masaryk University in Brno, Kamenice 753/55, 62500 Brno, Czech Republic

**Keywords:** DDH, gait analysis, foot posture, FPI-6, conservative treatment, hip surgery

## Abstract

Background: Developmental dysplasia of the hip (DDH) is recognized as a leading cause of significant long-term complications, including inaccurate gait patterns, persistent pain, and early regressive joint disorder, and it can influence families functionally, socially, and psychologically. Methods: This study aimed to determine foot posture and gait analysis across patients with developmental hip dysplasia. We retrospectively reviewed participants referred to the pediatric rehabilitation department of KASCH from the orthopedic clinic between 2016 and 2022 (patients born 2016–2022) with DDH for conservative brace treatment. Results: The foot postural index for the right foot showed a mean of 5.89 (*n* = 203, SD 4.15) and the left food showed a mean of 5.94 (*n* = 203, SD 4.19). The gait analysis mean was 6.44 (*n* = 406, SD 3.84). The right lower limb mean was 6.41 (*n* = 203, SD 3.78), and the left lower limb mean was 6.47 (*n* = 203, SD 3.91). The correlation for general gait analysis was r = 0.93, presenting the very high impact of DDH on gait. Significant correlation results were found between the right (r = 0.97) and left (r = 0.25) lower limbs. Variation between the right and left lower limb *p*-values was 0.88 (*p* < 0.05). DDH affects the left lower limb more than the right during gait. Conclusion: We conclude that there is a higher risk of developing foot pronation on the left side, which is altered by DDH. Gait analysis has shown that DDH affects the right lower limb more than the left. The results of the gait analysis showed gait deviation in the sagittal mid- and late stance phases.

## 1. Introduction

Developmental dysplasia of the hip (DDH) is a common disorder associated with significant impairment of the hip joint [1,2]. Early recognition of DDH can prevent malformation and enhance the appropriate anatomy of the hip, thereby leading to recovery without the need for surgery [3].

The prevalence rate for DDH varies within countries and continents from 1–34/1000 (cases per birth) [4,5,6]. In 2020, Sadat-Ali [7] published 10.46/1000 cases per birth as the latest prevalence of DDH in the Kingdom of Saudi Arabia (KSA) [7]. However, the incidence may vary in specific areas such as the Middle East, the gulf area, and Italy [4,5,6,7].

The three leading pediatric hip conditions (PHCs) are Perthes, Developmental Dysplasia of the Hip (DDH), and Slipped Upper Femoral Epiphyses (SUFE), which are all perceived as causes of early degenerative hip osteoarthritis in adults [7,8]. If DDH is not diagnosed and treated early, the long-term outcomes of late treatment lead to unfavorable consequences [9]. Late-diagnosed or persistent DDH is increasingly recognized as a leading cause of significant long-term morbidity, including: pathological gait pattern, chronic pain, and early regressive joint disease, and can affect families functionally, socially, and psychologically [2,9].

A hip shift is a common complication in children with a neurology diagnosis, which can lead to hip dislocation [10]. This shift can have a negative effect on a range of motion, motor abilities, and personal care skills and can be linked to pain [9]. Children typically are born with normal anatomic hip alignment [1,2]. While growing, hip development may be afflicted for various reasons. In combination with muscle inequality and spasticity, decreased weight bearing has often been raised as the primary contributor to hip displacement [10]. It can decrease the femoral head’s contact with the acetabulum, leading to a reduced range of motion and bone deformities such as coxa valga, femoral anteversion, and acetabular dysplasia [1,9,10].

If DDH is diagnosed early and treated accordingly and constantly, the treatment outcomes are positive. Any incorrect use of rigid orthosis can cause complications such as avascular necrosis of the femoral head (AVN) or lead to paralysis of the femoral and obturator nerves. The need to immobilize the child’s legs to restrain active lower limb movement raises inquiry among parents and physicians, as they fear it may cause a delay in the child’s gross motor development, especially sitting, standing, and walking [1,10].

Pediatric foot alignment has been described with footprint assessments in many studies. All through early development, children’s feet develop a medial longitudinal arch, which is different from the adult community. Children’s foot posture must be assessed in every developmental stage with the presence/absence of fundamental elements—hypotonia or hypermobility [11].

In the available literature, we found a limited number of analyses concerning the influence of applying an abduction brace on gross motor development in children with DDH. The latest was published by Zgoda et al., 2010 [1].

This study aimed to determine the foot posture and gait analysis patterns across patients with developmental hip dysplasia receiving brace treatment. The main hypothesis for this study, congenital dislocation of the hip affects the foot and lower limb position unilaterally.

## 2. Materials and Methods

### 2.1. Study Area

This study was conducted at KASCH of the Ministry of National Guard Health Affairs (MNGHA), Riyadh, Kingdom of Saudi Arabia (KSA). KASCH is the nation’s most advanced children’s hospital and the first medical referral institute that served pediatric patients in the KSA.

### 2.2. Study Design

Patients referred to the pediatric rehabilitation department of KASCH from the orthopedic clinic between 2016 and 2022 (patients born 2016–2022) with DDH for conservative treatment were screened as Acute cases (Category A). 

The principal investigator gathered demographic information from the Best Care medical system and a family member, the parent. The results had been passed on for statistical interpretation.

Cohort study: We retrospectively collected all patients from the Pediatric Physiotherapy Department in KASCH receiving conservative treatment for DDH. All individuals were sampled, and information about their past assessments and investigations were collected.

### 2.3. Sampling Technique

Demographic information were collected and entered into the working sheet. Inclusion and exclusion criteria were applied to clear data according to our project description. The principal investigator prepared patient lists with phone numbers, and the co-investigators contacted all parents to check if they were willing to participate in our project and bring their child to the Pediatric Physiotherapy Outpatient Clinic at KASCH for Zebris gait and foot assessment. The physiotherapy and orthopedic consultant who participated in our project as co-investigators renewed physiotherapy referrals for patients if they were discharged from our services.

#### Participants

This study was carried out in the gait analysis laboratory at King Abdullah Specialized Children’s Hospital (KASCH), Riyadh, KSA. We called patients retrospective from 2016–2022. Parents signed a written consent form approved by the ethics commission.

Inclusion criteria: could independently walk for ten steps, were diagnosed with DDH by an orthopedic physician, and received a physiotherapy referral for abduction braces treatment.

Exclusion criteria: could not walk for ten steps, were diagnosed with DDH.

### 2.4. Protocol Tools

The foot posture was assessed barefoot, in a relaxed standing position using the standard protocol for the Foot Postural Index 6 (FPI-6).

Gait analysis was carried out in the gait laboratory under the supervision of the principal investigator, barefoot, on a hard floor, for at least ten steps.

The FPI-6 evaluates the multi-segmental nature of foot posture in all three planes and does not require specialized equipment. Each item of the FPI-6 scores between minus two and plus two, with a total of six items referring to positions of the forefoot, mid-foot and hind-foot, and the three planes of motion:

➢ talar head palpation;
➢ the symmetry of the supra- and infra-lateral malleolus curvature;
➢ inversion/eversion of the calcaneus;
➢ prominence in the region of the talonavicular joint;
➢ height of the medial longitudinal arch;
➢ abduction/adduction of the forefoot.

The FPI-6 score spectrum goes from minus twelve—highly supinated to plus twelve—highly pronated [12].

The Wee Glasgow Gait Index (WeeGGI) is a gait analysis and screening tool for the many clinicians who do not have access to gait laboratories and special equipment to benefit their clinical decision-making [13].

The WeeGGI focuses on eleven gait domains, designed by the neuro-biomechanics team at Westmark, Glasgow, which are the most compatible with a normal gait. The clinician presents a diagram for each parameter with a choice of three stick figures, each of which has explicit descriptors and or values:

➢ 0 for the standard or neutral position—indicates the ideal position for the joint or segment at the specific point in the gait sequence;
➢ 1 for mild deviation—indicates a mild deviation from the expected position;
➢ 2 for gross deviation—indicates a gross deviation.

A score applies to each outcome, and a single grade must be selected for each parameter. The score for all eleven parameters should be estimated. The higher the score, the higher the standard gait alteration, which leads to a higher indication for physiotherapy intervention [12,13].

### 2.5. Ethical Approval

The study was conducted according to the Declaration of Helsinki and was approved by the Institutional Review Board from KAIMRC, Riyadh, KSA, with memo Ref. No. IRBC/1747/21, study No. SP21R/364/06 on 23 August 2021 and by The Masaryk University Research Ethics Committee, Brno, Czechia, Ref. No. EKV-2021-018, proposal No. 0107/2021 on 31 May 2021.

### 2.6. Statistical Methods

The statistical analysis was conducted using TIBCO Statistica™ 14.0.1 (StatSoft, EN version, San Diego, CA, USA). For assessing the normality of distribution, we applied the Shapiro-Wilk test. To estimate the intergroup differences in the non-parametric distribution, we applied the Mann–Whitney test. The differences at *p* < 0.05 were defined as statistically significant.

## 3. Results

In our population study of 203 children, the mean age was 20.09 months, ranging from 11 (2.5%) to 132 (0.5%) months. All patients were referred to the physiotherapy department and diagnosed by orthopedic physicians with DDH for treatment with abduction braces. This included 143 patients treated conservatively only with abduction braces (70.4%), 32 presented after surgical hip reduction and with casting (15.7%), were referred for abduction braces after cast removal, and 28 children who were seen for brace treatment with another diagnosis (13.7%), such as neurology, rheumatology, ophthalmology, cardiac, and other orthopedic diagnoses. The study population gender distribution was 165 females and 38 males. We evaluated 406 feet and lower limbs in this study. All patients were assessed for foot posture using FPI-6 and gait analysis using the WeeGGI index (Table 1).

**Figure 1:**
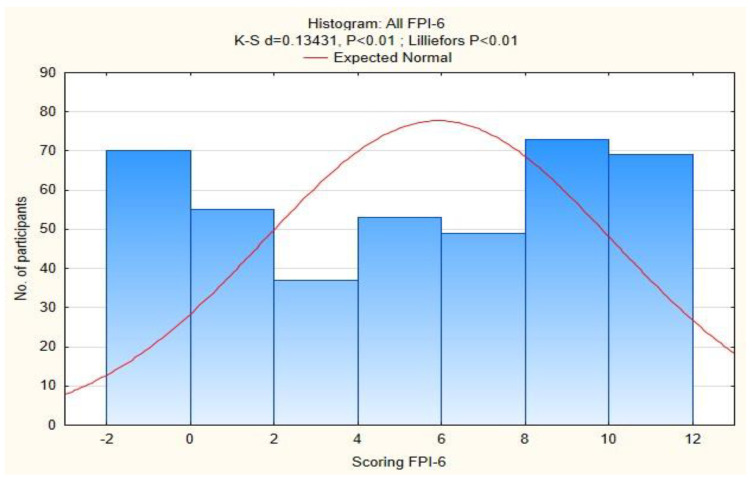
Histogram of all FPI-6 analyses.

The FPI-6 mean score for *n* = 406 feet was 5.9 (SD 4.16). Pronation is considered when FPI ≥ +6 and standard foot position is FPI < 6. Our group presented with pronation of both feet at 58.12% (236) and standard foot position had 41.87% (170) (Figure 1). Generally, our study group was at high risk of developing foot pronation. These results also show the difference between the right and left foot. The FPI-6 for the right foot showed a mean of 5.89 (*n* = 203, SD 4.15) and the left foot showed a mean of 5.94 (*n* = 203, SD 4.19) (Table 1). We concluded that there is a higher risk of developing foot pronation on the left side, which is altered by DDH. Foot supination (FPI ≥ −1) was not detected.

**Figure 2:**
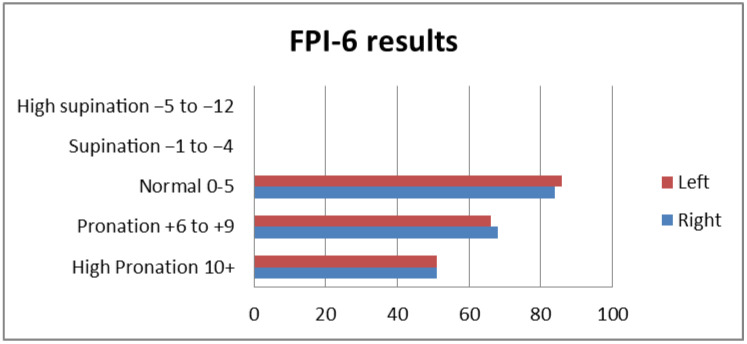
FPI-6 results from the right and left foot.

Right foot pronation with a score of FPI ≥ +6 was observed in 58.6% (119, *n* = 203) of children, normal foot position of FPI < 6 was observed in 41.3% (84, *n* = 203), and supination of FPI ≥ −1 was not detected in any participants. For the left foot, a normal position of FPI < 6 was observed in 42.3% (86, *n* = 203) of children, pronation was observed in 57.6% (117, *n* = 203), and supination of FPI ≥ −1 was not detected (Figure 2).

**Figure 3:**
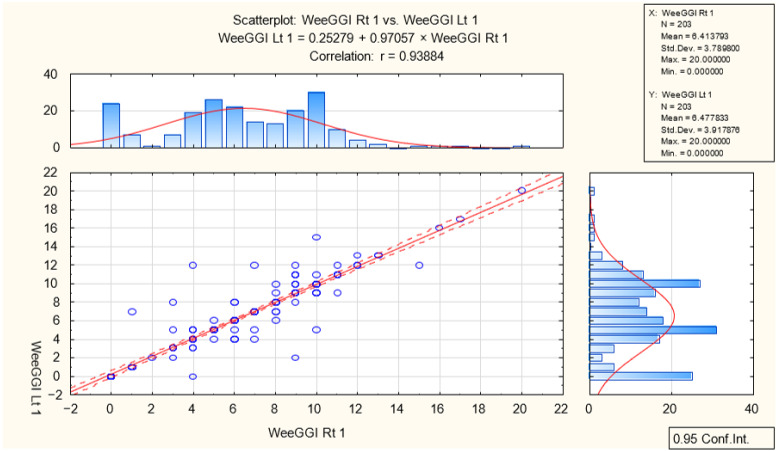
Correlation dialog between the right and left lower limbs.

WeeGGI gait analysis was measured during the same session as the foot postural index; patients independently walked barefoot on the hard floor for at least ten steps (Table 1). The WeeGGI mean was 6.44 (*n* = 406, SD 3.84), which can be considered a deviation from the regular gait pattern. The right foot mean score was 6.41 (*n* = 203, SD 3.78), and the left foot mean score was 6.47 (*n* = 203, SD 3.91). The correlation for general gait analysis was r = 0.93, presenting very high impact of DDH on gait. Significant correlation was found between the right r = 0.97 and left r = 0.25 lower limbs. Gait analysis showed that DDH affects the right lower limb more than the left. The sign test revealed difference between the right and left lower limbs with a *p*-value of 0.88 (*p* < 0.05). DDH affects the left lower limb more than the right during gait (Figure 3).

### Confounding Results

Additional statistical analyses were completed for conservative treatment with abduction braces and surgical treatment of DDH. Testing for foot posture and gait, the H0 (H0: There will be a difference in the foot posture and gait analysis between the conservative and surgical treatment of DDH) foot posture/gait was the same for conservative and surgical treatment of DDH. This study contained 143 patients who were treated conservatively, aged from 11 to 48 months, with a mean age of 16.22 months. The gender distribution was 31 (17.7%) males and 144 (82.2%) females. Post-hip surgery, 32 patients agreed to enter this study. The mean age was 19.6, the highest age was 48 and younger patient was 11 months old. We compared WeeGGI and FPI-6 results for the right and left limbs between the conservative treatment group and the post-hip-surgery participants (Table 2).

FPI-6 results showed no difference between the brace and operative DDH treatments (*p* < 0.05). The mean for the right foot in the conservative treatment group was 5.21 (SD 4.07, *n* = 143), while the mean in the surgery group was 7.25 (SD 4.11, *n* = 32). The mean for the left foot in the conservative treatment group was 5.25 (SD 4.14), while the mean in the surgery group was 7.28 (SD 4.04). A result of FPI-6, indicate foot pronation, in participants after surgical treatment for DDH (Table 2).

We tested each part of the eleven domains of WeeGGI, gait analysis. There was a significant difference in two WeeGGI gait domains of the right leg and four parts of the analysis on the left leg. Marked tests are significant at *p* < 0.05.

The right lower limb showed diversity in the:

- sagittal mid-stance on the right ankle, with a mean of 0.39 (SD 0.73) in the conservative treatment group and a mean of 0.50 (SD 0.67) in the surgery treatment group, and a *p*-value of 0.02;
- sagittal late stance thigh attitude, with a mean of mean 0.51 (SD 0.79) in the conservative treatment group and a mean of 0.65 (SD 0.90) in the surgery treatment group, and a *p*-value of 0.01.

The left lower limb showed diversity in the:

- sagittal initial contact knee analysis, with a mean of 0.55 (SD 0.49) in the conservative treatment group and a mean of 0.78 (SD 0.49) in the surgery treatment group, and a *p*-value of 0.03;
- sagittal mid-stance on the left ankle, with a mean of 0.39 (SD 0.73) in the conservative treatment group and a mean of 0.56 (SD 0.80) in the surgery treatment group, and a *p*-value of 0.01;
- sagittal mid-late stance shank attitude, with a mean of 0.48 (SD 0.76) in the conservative treatment group and a mean of 0.68 (SD 0.89) in the surgery treatment group, and a *p*-value of 0.03;
- sagittal late stance thigh attitude, with a mean of 0.43 (SD 0.49) in the conservative treatment group and a mean of 0.59 (SD 0.49) in the surgery treatment group, and a *p*-value of 0.01.

Results of the gait analysis showed gait deviation in the sagittal mid- and late stance phases.

## 4. Discussion

This project has shown the effect of DDH on gait and foot posture in pediatric patients after conservative treatment. The foot posture showed difference between conservative and surgical treatment of DDH. It is crucial to correct pathology in the hip, in this case, DDH, which affects gait and foot posture on the opposite leg due to the possibility of shifting body weight to create a higher burden on a healthy lower hip and foot.

It is commonly claimed that children with DDH who have not been treated start walking slightly later than healthy children [1]. This delay is about 2–3 months and usually does not exceed the average age of children starting to walk [14]. Dunn estimated that 20% of children with undiagnosed and untreated DDH do not start walking until 18 months [15]. Kamath and Bennet have demonstrated that the mean walking age in children not treated for this disorder was 13 months (with range of 9.5–18 months), which was, on average, one month later than in healthy children [16]. Zgoda et al.’s outcomes demonstrate that the abduction brace treatment does not appear to affect the child’s gross development. Though the three-week delay in walking is statistically significant, it does not influence their further locomotor development [1]. This study did not analyze the age when the child started to walk. We had patients starting DDH treatment at different age groups; the mean of the gait analysis was 20.09 months in the leading group and 16.22 months in the confounding group, which is lower than Dunn’s 1990 finding of 18 months but higher than Zgoda’s finding of 13 months. The youngest patient was 11 months old [1,15]. Gait analysis should be the golden standard for DDH patients. The visual gait analysis is now added as an outcome measure for all patients with DDH.

Reduced hip range of motion while sitting, standing, and walking is ordinary. In 2014, Larnert et al. defined an association between hip dislocation and pelvic obliquity, windswept deformity, and scoliosis [17]. Our study showed no difference or pathology in the coronal mid-stance pelvic obliquity.

In 2019, Gijon-Nogueron et al. published an article describing a significant difference (*p* < 0.01) between countries [11]. The mean for Spanish children was FPI = 4.00 (2.9), the mean for UK children was FPI = 4.9 (3.3), and the mean for Australian children was FPI = 4.7 (3.1). The general trend showed FPI scores declining in some countries [11]. The Saudi population in our study had a mean of FPI = 5.92 (4.16), which is higher than the average stated in Gijon-Nogueron et al.’s publication. The opportunity to access average FPI scores empowers clinicians to inform parents of what is ‘average’ and what is ‘normal’ at any age, it has been statistically proven [11]. After this project, the FPI-6 was uploaded to the pediatric physiotherapy outcome measures at KASCH.

Wenger et al. found that the percentage of high dislocations was 5.5% for children diagnosed in their first year, 31% when diagnosed in the second year, and 67% when diagnosed after the second year, showing that hip dislocations worsened with time [18]. As soon as the hip reduction is associated, there is a lower risk of avascular necrosis of the femoral head after treatment [18,19]. Our study had one female (0.49%) with AVN complications resolving in Perthes.

Patients screened and diagnosed early and treated conservatively have a success rate of 90%. Within Saudi Arabia, there is a wide-ranging variability of presentations from newborn to six years of age, making treatment decisions difficult and outcomes unpredictable [7].

In our practice, the physiotherapist receives patients for abduction braces treatment as the first or second choice after the Pavlik harness or surgical intervention. According to Mulpuri et al., some studies revealed that the initial treatment method was splinting, with discrepancies between the younger and older groups regarding surgical management [20]. Some studies of limited strength have examined the use of rigid and soft splints; however, the results are inconsistent with small effect sizes [21].

With our project regarding the prevalence and outcomes of DDH in physiotherapy practice, we want to highlight the importance of rehabilitation, as Jennings et al. wrote in 2016: “*DDH is a poorly presumed disorder as evidenced by the profusion of literature, both recent and historical*.” [2].

The main limitations of this study were lack of probability, different physiotherapists with variable years of experience assessing participants’ gaits and foot posture, child presentation during analysis.

## 5. Conclusions

We concluded that there is a higher risk of developing foot pronation on the left side, which is altered by DDH. Gait analysis has shown that DDH affects the right lower limb more than the left. The results of the gait analysis showed gait deviation from the regular gait pattern in the sagittal mid- and late stance phase.

## Figures and Tables

**Table 1:** Descriptive statistics.

Variable	*N*	Mean	Minimum	Maximum	Std.Dev.
Age	203	20.09	11.00	132.00	17.37
FPI-6 all	406	5.91	0.00	12.00	4.16
FPI-6 Right	203	5.89	0.00	12.00	4.15
FPI-6 Left	203	5.94	0.00	12.00	4.19
WeeGGI all	406	6.44	0.00	20.00	3.84
WeeGGI Right	203	6.41	0.00	20.00	3.78
WeeGGI Left	203	6.47	0.00	20.00	3.91

**Table 2:** Foot and gait analysis results for the conservative treatment and surgery groups.

Variables	Valid *N*	Mean	Minimum	Maximum	Std. Dev.
FPI-6 general score conservative Rt	143	5.21	0.00	12.00	4.07
FPI-6 general score surgery Rt	32	7.25	0.00	12.00	4.11
FPI-6 general score conservative Lt	143	5.25	0.00	12.00	4.14
FPI-6 general score surgery Lt	32	7.28	0.00	12.00	4.04
WeeGGI general score conservative Rt	143	5.76	0.00	12.00	3.55
WeeGGI general score surgery Rt	32	7.21	0.00	13.00	2.80
WeeGGI general score conservative Lt	143	5.65	0.00	12.00	3.65
WeeGGI general score surgery Lt	32	7.78	0.00	13.00	3.01

## Data Availability

V.V. had full access to the data for this study and took responsibility for the integrity of the data and the accuracy of data analysis.
